# *QTug.sau-3B* Is a Major Quantitative Trait Locus for Wheat Hexaploidization

**DOI:** 10.1534/g3.114.013078

**Published:** 2014-08-15

**Authors:** Ming Hao, Jiangtao Luo, Deying Zeng, Li Zhang, Shunzong Ning, Zhongwei Yuan, Zehong Yan, Huaigang Zhang, Youliang Zheng, Catherine Feuillet, Frédéric Choulet, Yang Yen, Lianquan Zhang, Dengcai Liu

**Affiliations:** * Triticeae Research Institute, Sichuan Agricultural University at Chengdu, Wenjiang, Sichuan 611130, People’s Republic of China; † Institute of Ecological Forestry, Sichuan Agricultural University at Chengdu, Wenjiang, Sichuan 611130, People’s Republic of China; ‡ Key Laboratory of Adaptation and Evolution of Plateau Biota, Northwest Institute of Plateau Biology, Chinese Academy of Sciences, Xining 810001, People’s Republic of China; § INRA University Blaise Pascal, Joint Research Unit 1095 Genetics Diversity and Ecophysiology of Cereals, Clermont-Ferrand 63039, France; ** Department of Biology and Microbiology, South Dakota State University, Brookings, South Dakota 57007

**Keywords:** allopolyploidy, *CYCA1;2/TAM*, first division restitution, unreduced gametes, *Triticum aestivum*

## Abstract

Meiotic nonreduction resulting in unreduced gametes is thought to be the predominant mechanism underlying allopolyploid formation in plants. Until now, however, its genetic base was largely unknown. The allohexaploid crop common wheat (*Triticum aestivum* L.), which originated from hybrids of T. turgidum L. with *Aegilops tauschii* Cosson, provides a model to address this issue. Our observations of meiosis in pollen mother cells from *T. turgidum*×*Ae. tauschii* hybrids indicated that first division restitution, which exhibited prolonged cell division during meiosis I, was responsible for unreduced gamete formation. A major quantitative trait locus (QTL) for this trait, named *QTug.sau-3B*, was detected on chromosome 3B in two *T. turgidum*×*Ae. tauschii* haploid populations. This QTL is situated between markers *Xgwm285* and *Xcfp1012* and covered a genetic distance of 1 cM in one population. *QTug.sau-3B* is a haploid-dependent QTL because it was not detected in doubled haploid populations. Comparative genome analysis indicated that this QTL was close to *Ttam-3B*, a collinear homolog of *tam* in wheat. Although the relationship between *QTug.sau-3B* and *Ttam* requires further study, high frequencies of unreduced gametes may be related to reduced expression of *Ttam* in wheat.

Allopolyploidy, defined as the presence of two or more different genomes, is prevalent among many groups of plants (Stebbins 1950; Grant 1971). Allopolyploid plants are common in natural ecosystems and among important crop species, such as bread wheat, durum wheat, oat, cotton, sugarcane, canola, coffee, and tobacco (Udall and Wendel 2006; Soltis *et al.* 2009). They usually originate by means of interspecific or intergeneric hybridization followed by spontaneous doubling of chromosome numbers. Wide hybridization brings divergent genomes from different species together in amphihaploid (analogous to haploid) F_1_ hybrids. Chromosome doubling of F_1_ hybrids generates amphiploids (allopolyploids), which stabilizes the reproductive cycle because it confers bivalent chromosome pairing and fertility.

Unreduced gametes have the same number of chromosomes as somatic cells. Formation of unreduced gametes is believed to be the most important mechanism for chromosome doubling in wide hybrids (Harlan and De Wet 1975; Ramsey and Schemske 1998, 2002). In most cases, unreduced gametes in hybrids result from meiotic restitution, in which meiotic cell division is converted into a mitosis-like nonreductional process, *i.e.*, meiotic nonreduction, which generates dyads instead of the normal tetrads at the end of meiosis (Bretagnolle and Thompson 1995; Lyrene *et al.* 2003; Ramanna and Jacobsen 2003; Fawcett and Van De Peer 2010; De Storme and Geelen 2013). Both first division restitution (FDR) and second division restitution are associated with meiotic restitution. However, the production of unreduced gametes in wide hybrids usually results from FDR (Ramanna and Jacobsen 2003). During standard meiosis in a wide hybrid, chromosomes are typically unpaired, univalents usually migrate randomly to the poles, and dyads subsequently form in meiosis I; the second division with sister chromatid separation proceeds, usually forming tetrads and reduced gametes in meiosis II (Oleszczuk and Lukaszewski 2014). However, in FDR, univalents separate sister chromatids in anaphase I, and there is no second division (Ramanna and Jacobsen 2003; Oleszczuk and Lukaszewski 2014). Until now, the genetic basis for meiotic restitution in wide hybrids was largely unknown, although some genes have been identified from the diploid model plant *Arabidopsis thaliana* (De Storme and Geelen 2011; Brownfield and Köhler 2011; Wijnker and Schnittger 2013).

Bread wheat (Triticum aestivum L., 2n = 6x = 42, AABBDD) is an important cereal grain crop that provides nearly 20% of the calories and protein for humankind (Hawkesford *et al.* 2013). It is a classic example of speciation via allopolyploidization, originating from the natural hybridization of *T. turgidum* L. (2n = 28, AABB) and *Aegilops tauschii* Cosson (2n =14, DD) (McFadden and Sears 1946; Kihara and Lilienfeld 1949), followed by spontaneous genome doubling via unreduced gametes (Cai and Xu 2007; Jauhar 2007). Unreduced gametes have been observed frequently in F_1_ hybrids of *T. turgidum*×*Ae. tauschii* (Zhang *et al.* 2010) as well as other Triticeae species (Matsuoka 2011; Silkova *et al.* 2011), and in haploid plants of *T. turgidum* (Jauhar 2003). This phenomenon is caused by FDR or other single-division meiotic events (Xu and Joppa 1995, 2000; Matsuoka and Nasuda 2004; Zhang *et al.* 2007). Although *Ae. tauschii* has some effect (Zhang *et al.* 2010; Matsuoka *et al.* 2013), *T. turgidum* genotypes are considered to play the most important role in meiotic restitution (Fukuda and Sakamoto 1992; Xu and Dong 1992; Jauhar 2003). *T. turgidum* ssp. durum cultivar Langdon (LDN) is one of the genotypes that is most studied for meiotic restitution. Several studies have consistently indicated that LDN produces a high frequency of unreduced gametes in hybrids with *Ae. tauschii* (Xu and Joppa 2000; Matsuoka and Nasuda 2004; Zhang *et al.* 2008), and that the phenomenon is controlled by major genes (Xu and Joppa 1995). However, cytological analysis of a set of LDN D-genome disomic substitution lines failed to locate the causal genes, because some D-genome chromosomes poorly compensated for their homeologous A-genome or B-genome counterparts that carry factors affecting meiotic restitution (Xu and Joppa 2000; Zhang *et al.* 2008).

Meiotic restitution and unreduced gametes do not occur in normal tetraploid and hexaploid wheat, which have diploid-like meiotic behavior, *i.e.*, bivalent pairing. However, they occur in polyhaploids and intergeneric hybrids involving wheat and the related species, in which asynapsis results from the absence of homologous chromosome (Jauhar 2003, 2007; Cai *et al.* 2010; Silkova *et al.* 2012). Asynapsis is the key feature of unreduced gamete formation (Wang *et al.* 2010; Ressurreição *et al.* 2012; Silkova *et al.* 2013); therefore, it is referred to as "univalent-dependent meiotic nonreduction" (De Storme and Geelen 2013).

*T. turgidum*×*Ae. tauschii* triploid hybrids that show a very low level of chromosome pairing provide a desirable background for observation of meiotic restitution. Under these circumstances, the selfed seed set rate is a good indicator for the production of functionally unreduced gametes (Matsuoka and Nasuda 2004; Dewitte *et al.* 2012; Matsuoka *et al.* 2013). Here, we report the use of two *T. turgidum*×*Ae. tauschii* triploid populations to map a major quantitative trait locus (QTL) affecting hexaploidization in wheat. A collinear homolog of *cyca1;2/tam*, which results in the formation of unreduced gametes in *Arabidopsis thaliana* (d’Erfurth *et al.* 2010), was found to be closely located to the identified QTL in wheat and thus was further analyzed.

## Materials and Methods

### Production of hybrids and cytological observations

LDN and *T. turgidum ssp. turgidum* lines AS313 and AS2255 were pollinated by *Aegilops tauschii ssp. tauschii* accession AS60, as previously described by Zhang *et al.* (2007). No embryo rescue technique or hormone treatment was applied for the production of the wide hybrids. The triploid hybrids were germinated in Petri dishes and then transplanted in the field. F_2_ seeds were obtained by selfing the F_1_ triploids.

Observations on chromosome numbers in root-tip cells of individual plants and meiosis in pollen mother cells (PMCs) in individual anthers of the hybrids were performed according to procedures previously described by Zhang *et al.* (2007). Genomic *in situ* hybridization (GISH) and fluorescence *in situ* hybridization (FISH) were performed as previously described by Hao *et al.* (2011, 2013). Briefly, to distinguish A, B, and D genomes by GISH, samples of total genomic DNA of *T. urartu* and *Ae. tauschii* were labeled with biotin-16-dUTP (Roche Diagnostics Gmbh, Mannheim, Germany) and digoxigenin-11-dUTP (Roche), respectively. Unlabeled genomic DNA from *Ae. speltoides* was used as blocking DNA. To identify the chromosome constitutions of root-tip cells, clones pAs1 (Nagaki *et al.* 1995), pSc119.2 (Contento *et al.* 2005), and pTa71 (Fujisawa *et al.* 2006) were used as probes for FISH. To observe centromeres during meiosis, the primer set (6C6-3-F1: 5′-CTACTTCCACTGCACCAGAC-3′; 6C6-3-R1: 5′-CGCCCTACTTTGCACACAAAA-3′; Supporting Information, Table S1), designed according to the centromeric sequence 6C6-3 (Zhang *et al.* 2004), was used to generate the probe by PCR, which was then labeled with digoxigenin-11-dUTP (Roche) by nick translation, according to the manufacturer’s instructions. An Olympus BX-51 microscope coupled to a Photometric SenSys Olympus DP70 CCD camera was used to observe and document the chromosomes. The raw images were processed using Photoshop v. 7.1 (Adobe Systems Incorporated, San Jose, CA).

### Data collection and QTL mapping

**Figure 1:**
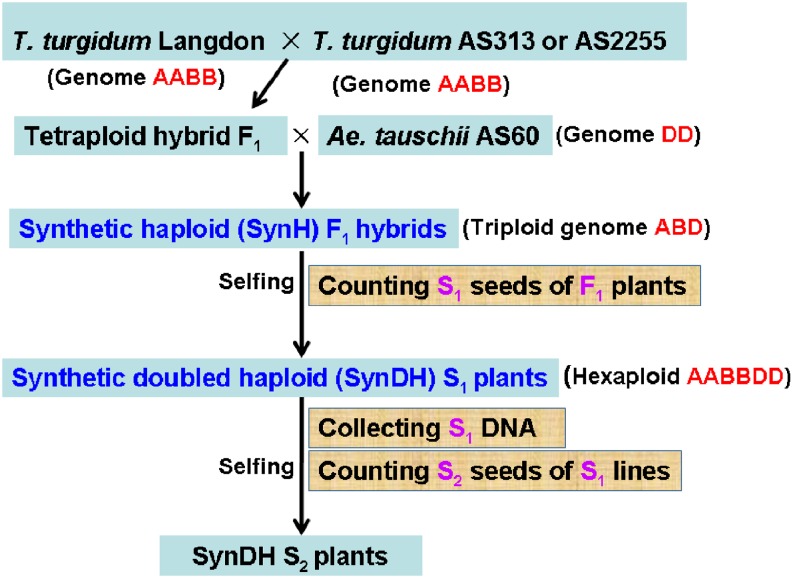
Outline of the production of haploid and doubled haploid populations.

The populations used for QTL mapping were previously produced (Zhang *et al.* 2011). In brief (Figure 1), diploid AS60 was used to pollinate tetraploid LDN×AS313 and LDN×AS2255 F_1_ hybrids to form triploid F_1_ hybrid populations SynH1 and SynH2, respectively, which were then self-pollinated to produce doubled haploid populations SynDH1 and SynDH2 by spontaneous chromosome doubling. These mapping populations have recombinant A and B chromosomes from the *T. turgidum* parents in a background of nonrecombinant D chromosomes from *Ae. tauschii*. All above plant materials were grown at the experiment farm of the Triticeae Research Institute of Sichuan Agricultural University. The F_1_ seeds were germinated in Petri dishes before being transplanted into the field. Individual plants were spaced 10 cm apart within 2-m-long rows; the row spacing was 30 cm.

All mature spikelets were harvested from individual hybrid plants. The numbers of seeds in the first and second florets of each harvested spikelet were scored. Selfed seed numbers were used as an indicator of hybrid genome doubling (Matsuoka *et al.* 2013). For an individual triploid F_1_ plant, the selfed seed set rate was calculated as the ratio of number of seed set over the number of florets examined. For each double haploid line, seed set rates of 10 F_2_ plants were obtained. A previous study indicated that approximately 20% of F_2_ (S_1_) plants were aneuploid (Zhang *et al.* 2011). To avoid the possible influence on the seed set rate by aneuploidy, only the values from the five plants with the highest seed set rates per line were averaged to obtain an overall score for that line.

The genetic map previously constructed for SynDH1 (Zhang *et al.* 2012) (Table S2) was used for QTL mapping. Nine SSR markers that had been mapped on chromosome 3B in that map also showed polymorphism in SynDH2 (Luo *et al.* 2012). They were also used to construct a genetic map of chromosome 3B (Table S3). QTL IciMapping v3.1 software (http://www.isbreeding.net), which is based on an inclusive composite interval mapping (ICIM-ADD) model (Li *et al.* 2007), was used for map construction and QTL analysis (Zhang *et al.* 2012). The Kosambi function was used to calculate genetic distances (Kosambi 1943).

The selfed seed set rates were used to identify QTL in the haploid and doubled haploid populations (Table S2 and Table S3). Threshold values were calculated using 1000 permutations with a 0.0500 type I error. The proportion of phenotypic variation explained by each QTL was calculated using single factor regression (R^2^).

### Cloning and sequencing the candidate gene for unreduced gametes

Marker collinearity between genomes of related species was used to predict syntenic loci for unreduced gametes. Rice gene Os01g13260.1 is a homolog of *TAM* (*Tardy Asynchronous Meiosis*)/*CYCA1;2* (A-type cyclin) in *Arabidopsis thaliana* (d’Erfurth *et al.* 2009). Based on its coding DNA sequence (CDS), a pair of primers (F1: 5′-GTCGCTGAAGAATATCGTCTTGTT-3′; R1: 5′-TGTTGGCTGCAGTATGAATTT-3′) was designed to amplify partial sequences of its homologs in LDN. The PCR mixture was prepared using EX Taq polymerase (TaKaRa Biotechnology Co., Ltd, Dalian, China) according to the manufacturer’s instructions. PCR amplification was performed in a GeneAmp PCR System 9700 (Applied Biosystems, Singapore) with the following conditions: 95° for 4 min, 35 cycles of 94° for 30 sec, 58° for 30 sec, and 72° for 1 min, followed by 72° for 10 min.

Full-length CDS of the *TAM* homologs were then amplified from *T. turgidum* lines LDN, AS313, AS2255, PI 14892, and AS308, and hexaploid bread wheat cultivar Chinese Spring with PCR primers F2: 5′-ATGTCGAGCAACTCCGC-3′ and R2: 5′-CTAGCATGCCGCGTCCC-3′. These *T. turgidum* lines differed in their abilities of forming functional gametes in hybrids with *Ae. tauschii* (Zhang *et al.* 2010). Total RNA samples from their roots were used in RT-PCR with PrimeSTAR HS DNA Polymerase in GC Buffer (TaKaRa) according to the manufacturer’s instructions. The PCR comprised 35 cycles of 98° for 10 sec, 60° for 5 sec, and 72° for 2 min. PCR products were separated on 1.2% agarose gels, purified using a Gel DNA Recovery Kit (PUEX, USA), and then cloned into a pMD19-T vector using a cloning kit from TaKaRa. Positive clones were identified and then sequenced by BGI (Beijing, China). At least eight clones were sequenced for each sample. Sequence alignments were performed using the DNAMAN 6.0 Demo software (Lynnon Biosoft).

### RNA extraction, cDNA synthesis, and quantitative real-time PCR

When flag leaves emerged to approximately 5 cm, individual spikelets were dissected from the rachis, and all three synchronized anthers within a spikelet were removed from the first floret. One anther was fixed in 1:3 (v/v) acetic acid:ethanol and stored at 4°. This anther was later squashed in 2% acetocarmine to determine the developmental stage under a light microscope. The two remaining anthers were collected in a 2-ml EP tube frozen in liquid nitrogen and stored at −80° until used. These two anthers at similar phases of the cell cycle constituted a sample as a biological replicate. After anthers in the EP tube that was placed on a EP tube plate with liquid nitrogen were carefully grinded into fine powder using a pre-cooled glass rod, the RNAprep Pure Plant Kit (TIANGEN, China) was used to isolate the total RNA according to the manufacturer’s instructions. The RNA samples (approximately 0.4–0.6 ug per sample) with high quality were chosen for the following analysis. The Primescript RT reagent Kit With gDNA Eraser (Takara) was then used to synthesize the first strand cDNA according the manufacturer’s instructions.

Quantitative RT-PCR (qRT-PCR) was performed in a 25-μl reaction that contained 12.5 μl SYBR Premix Ex Taq II (Takara), 2 μl cDNA solution or water (control), and 2 μl primers (10 µM). The primers (F10-RT: 5′-GCTTACCCTCCTTCACTTGT-3′; R10-RT: 5′-CCTTCACGCAATCGCATAG-3′) were designed according to sequences of wheat *TAM*. The wheat β-actin gene was used as the internal reference (Devisetty *et al.* 2010). Three technical repeats by three separate RT-PCR per sample and three biological replicates per treatment were performed in a Bio-Rad CFX96 RealTime PCR System (Bio-Rad, USA) by the following: 30 sec at 95°, followed by 40 cycles of 5 sec at 95°, 30 sec at 60°, and 30 sec at 72°. A melting curve was obtained from the product at the end of amplification by heating from 65° to 95°. The 2*^−ΔΔCt^* method was used to analyze the qRT-PCR data (Livak and Schmittgen 2001).

### Statistical analysis

Significant testing was performed using data analysis function of Microsoft Excel 2007. Correlation analyses were performed using regression model of Microsoft Excel 2007. The distribution maps of seed set ratio for SynH1 and SynH2 populations were generated by histogram model of SPSS 18.0.

## Results

### The capacity of hexaploidization by the *T. turgidum*×*Ae. tauschii* hybrids

F_1_ hybrid seeds were successfully produced when LDN, AS313, and AS2255 were crossed with AS60. All the analyzed F_1_ plants grew vigorously and had tough tenacious glumes, a trait obviously inherited from *Ae. tauschii*. They were partially fertile and produced F_2_ seeds by selfing. Although there was variation among plants within a same hybrid combination, the seed set rate of LDN×AS60 (4035/8748 or 46.12% over 11 plants, range from 26.09% to 65.38%) was significantly higher than for AS313×AS60 (396/2162 or 18.32% over 4 plants, range from 14.34% to 25.00%) (t = 3.61, *P* ≤ 0.01) and AS2255×AS60 (810/4802 or 16.87% over 6 plants, range from 12.17% to 26.29%) (t = 4.89, *P* ≤ 0.01).

**Figure 2:**
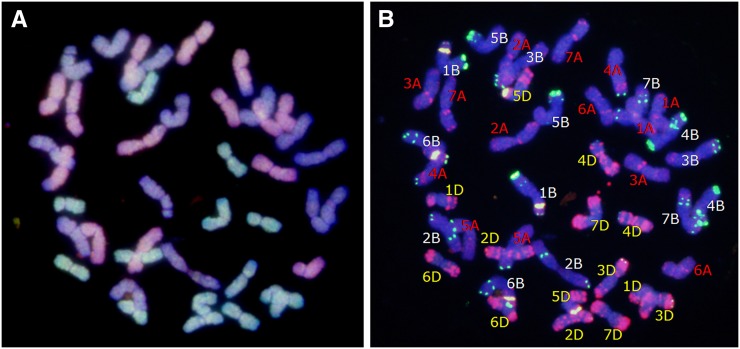
Chromosome constitutions. Genomic *in situ* hybridization on 42 root-tip chromosomes from A (pink), B (blue), and D (green) genomes (A). Fluorescence *in situ* hybridization using PAs1 (red), PSc119.2 (green), and PTa71 (yellow) as probes (B).

Randomly selected F_2_ seeds were analyzed for chromosome constitutions by root-tip chromosome counts, GISH (Figure 2A), and FISH (Figure 2B). Cytological analysis indicated that most of the analyzed F_2_ plants were euhexaploids with 42 chromosomes, indicating that the genomes in the F_2_ plants had spontaneously doubled. Aneuploids (2n = 40, 41, 43, or 44) were also present in the three hybrid combinations. However, the aneuploid frequency was different among the LDN×AS60 hybrids (13.2%, or 5 of 38 seeds), the AS313×AS60 hybrids (33.3%, or 3 of 9 seeds), and the AS2255×AS60 hybrids (33.3%, or 7 of 21 seeds). The combined aneuploid frequency for the AS313×AS60 and AS2255×AS60 hybrids was higher than that for LDN×AS60 hybrids (|u| = 1.98 > u_0.05_ = 1.96). These results confirmed our previous observation that LDN×AS60 hybrids have a higher capacity for hexaploidization than AS313×AS60 and AS2255×AS60 hybrids (Zhang *et al.* 2007, 2008, 2010).

### Male meiosis of *T. turgidum*×*Ae. tauschii* hybrids

**Figure 3:**
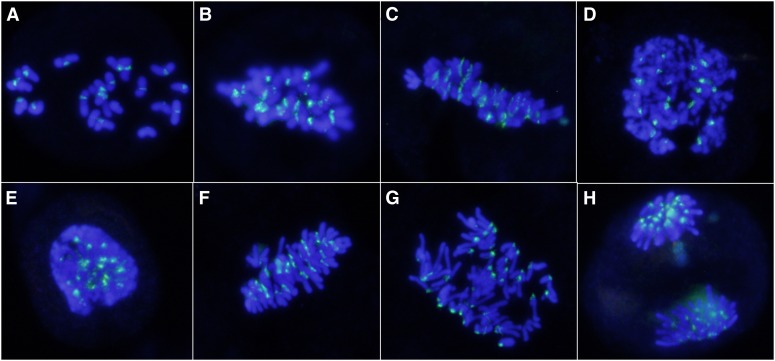
First division restitution (FDR) in LDN×AS60 F_1_ hybrids. Twenty-one univalents are visible at early metaphase (A). Univalents aligned on the equator at metaphase (B). Sister chromatids starting to separate (C). Univalents not aligned on the equator when they begin to split into sister chromatids remain connected at the centromeres (D). A restitution nucleus formed (E) and chromosomes subsequently congregate on the equator (F). Chromosomes undergoing equational division at anaphase (G, H). Centromeres were labeled in green.

**Figure 4:**
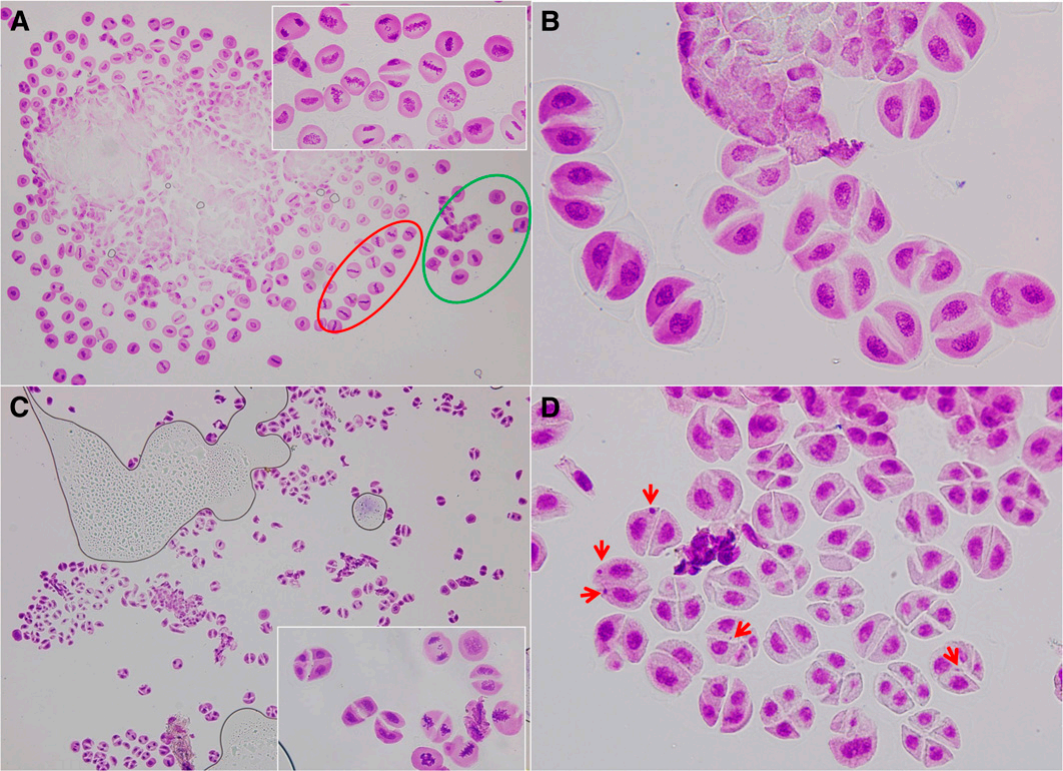
Final products of first division restitution (FDR) and standard meiotic division (SMD). FDR occurs in almost all PMCs of an anther from an LDN×AS60 hybrid (A) and produces dyads (B). Co-existence of FDR and SMD in an anther from an AS2255×AS60 hybrid (C) and resultant dyads and tetrads (D). Arrowheads in (D) indicate micronuclei. Adjacent cells (A) within an anther seem to be more synchronous than random cells (represented by red and green circles).

To investigate the reason for the difference in hexaploidization capacity between *T. turgidum*×*Ae. tauschii* hybrids, conventional staining and FISH using centromere probe 6C6-3 were used to observe meiosis in PMCs of the hybrids. At early metaphase I, the chromosomes generally appeared as univalents (Figure 3A), indicating that homeologous pairing was rare because of the presence of the Ph1 gene in *T. turgidum* (Okamoto 1957; Riley and Chapman 1958). In subsequent meiotic processes, we observed FDR and formation of dyads (Figure 3, B–H). The observed univalent behaviors among the analyzed PMCs of the three hybrid combinations suggest that FDR might have two pathways: (1) univalents were aligned on the equator at metaphase I (Figure 3B), followed by separation of sister chromatids (Figure 3C); and (2) univalents were not aligned on the equator and, when they began to split into sister chromatids, they remained connected at the centromeres (Figure 3D). They then formed a restitution nucleus (Figure 3E) and subsequently congregated on the equator (Figure 3F). Chromosomes underwent equational division at anaphase and dyad daughter cells were the only final products (Figure 3, G and H; Figure 4). In the LDN×AS60 hybrids, FDR predominated in all analyzed PMCs (Figures 4, A and B). FDR was also observed in the AS313×AS60 and the AS2255×AS60 hybrids; however, a large number of PMCs in the two hybrid combinations did not undergo meiotic restitution and produced triads and tetrads that might have undergone standard meiotic division (Figures 4, C and D).

**Figure 5:**
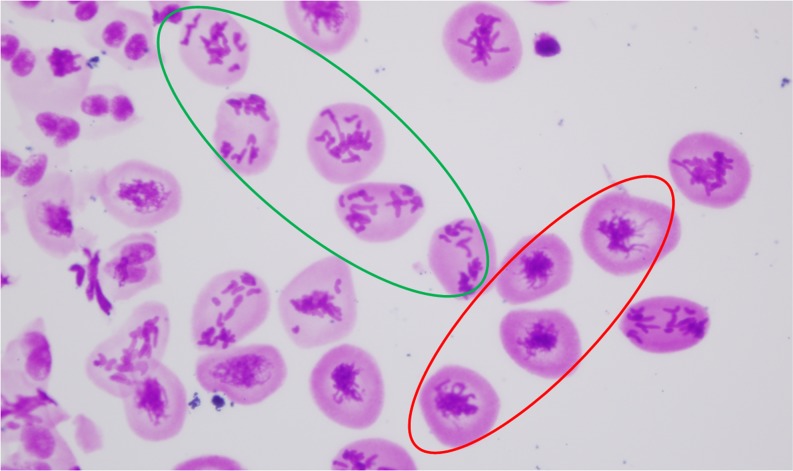
Asynchronous cell cycles from an anther of an AS2255×AS60 hybrid. Adjacent cells within an anther seem to be more synchronous (represented by red and green circles).

Asynchronous cell cycles were observed in all the analyzed hybrids. This asynchrony was observed regularly in the early metaphase stages (Figure 5), suggesting that PMCs entered metaphase at different time points. Figure 4C shows the coexistence of FDR and standard meiotic divisions at various stages from restitution nucleus to telophase II in AS2255×AS60 hybrids, whereas Figure 4A shows FDR with a narrower range of stages from restitution nucleus to telophase I in LDN×AS60 hybrids. The AS2255×AS60 hybrids appeared more asynchronous than the LDN×AS60 hybrids. Meanwhile, there was a trend that adjacent cells seemed to be more synchronous within an anther (Figures 4A, 5).

### Seed setting in *T. turgidum*×*Ae. tauschii* hybrids was closely related to dyad formation

Dyads are the final products of meiotic restitution. Therefore, we compared frequencies of dyads. LDN×AS60 hybrids mostly generated dyads (97.78%, 176/180), with few triads and tetrads (Table 1). The proportion of dyads in LDN×AS60 hybrids was significantly higher than in the AS313×AS60 (41.95%) (|u| = 11.50 > u_0.01_ = 2.58) and AS2255×AS60 (57.38%) (|u| = 9.95) hybrids. Moreover, LDN×AS60 hybrids showed a lower proportion of dyads with micronuclei (1.11%) than AS313×AS60 (12.07%) (|u| = 4.18 > u_0.01_ = 2.58) and AS2255×AS60 (20.90%) (|u| = 6.26) hybrids. This result indicates that Langdon is more capable of promoting meiotic restitution in F_1_ hybrids with *Ae. tauschii* AS60 than with AS313 and AS2255.

**Table 1:** Frequencies of cell types among three hybrid combinations

Hybrid Combination	Dyads			Triads			Tetrads			Polyads		
	A	B	Total	A	B	Total	A	B	Total	A	B	Total
Langdon×AS60	1.1%	96.7%	97.8%	0	1.1%	1.1%	0.6%	0.6%	1.1%	0	0	0
AS313×AS60	12.1%	29.9%	42.0%	4.0%	12.6%	16.7%	10.9%	29.9%	40.8%	0	0.6%	0.6%
AS2255×AS60	20.9%	36.5%	57.4%	1.6%	6.6%	8.2%	8.0%	25.6%	33.6%	0.2%	0.6%	0.8%

Column A represents the frequency of plants with a micronucleus; column B represents those without. The numbers of pollen mother cells were 180, 174, and 488 for the Langdon×AS60, AS313×AS60, and AS2255×AS60 hybrids, respectively.

We further analyzed the relationship between dyads and fertility of the triploid *T. turgidum*×*Ae. tauschii* hybrids. Dyad formation is expected to lead to fertility of hybrids. Usually, two methods are used to determine the fertility in hybrids. One is to observe the ratio of living and dead pollen grains by observing pollen gains stained with aceto-carmine. The other is to determine the selfed seed setting level. As Matsuoka *et al.* (2013) pointed out, variation in the size and staining intensity makes it difficult to evaluate the frequencies of functional pollen grains based on morphology in *T. turgidum*×*Ae. tauschii* hybrids. In the present study, selfed seed setting rates were used as measures of fertility. Correlation analysis indicated that the dyad ratio was positively correlated with the seed set ratio among the three hybrid combinations (R^2^ = 0.88), indicating that high fertility resulted from high-frequency dyad formation.

F_2_ seeds included euhexaploids and aneuploids. Euhexaploid F_2_ seeds resulted from the union of two unreduced euploid gametes, whereas aneuploids resulted from aneuploid gametes with missing or additional chromosomes. Dyads with micronuclei usually indicated the presence of aneuploids, and the proportion of dyads containing micronuclei was positively correlated with the proportion of aneuploid F_2_ seeds (R^2^ = 0.80). The results confirmed that the seed set on F_1_ plants resulted from the production of dyads produced by meiotic restitution.

### QTL analysis of hexaploidization capacity in the *T. turgidum*×*Ae. tauschii* haploids

**Figure 6:**
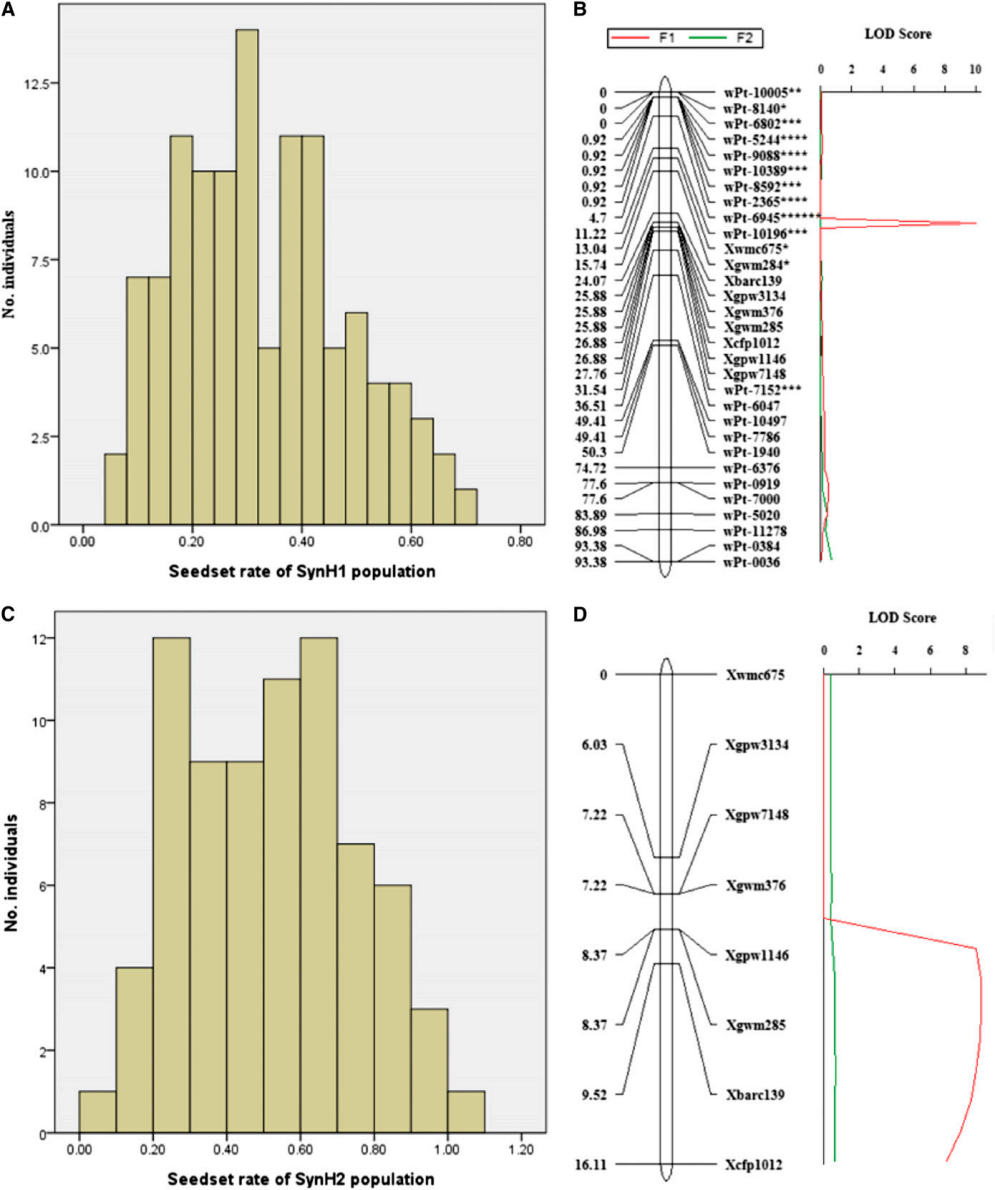
Frequency distribution and QTL for hybrid genome doubling. Frequency distribution of haploid plants with different seed setting rate for populations SynH1 (A) and SynH2 (C) and a QTL for the two haploid (F1) populations (B and D, red). This QTL was not detected in the doubled haploid (F_2_) populations (B and D, green). Asterisk (*) after marker indicates deviation from the 1:1 expected segregation ratio at *P* < 0.05, ** at *P* < 0.01, *** at *P* < 0.005, **** at *P* < 0.001, and ****** at *P* < 0.0001.

To further investigate the genetic basis of the high capacity of hexaploidization in LDN, haploid (triploid) population SynH1 (LDN/AS313//AS60) and its corresponding doubled haploid (hexaploid) population SynDH1 were used for QTL mapping (Figure 1). The molecular map constructed by the two populations should be the same, although the ploidy of the populations was different. The molecular data for the 113 SynDH1 lines were used to construct a linkage map containing 588 molecular markers that had been assigned to the 14 A-genome and B-genome chromosomes, covering a total genetic distance of 2,048.79 cM, with a mean distance of 3.48 cM between adjacent markers (Zhang *et al.* 2012). The selfed seed set rates of the SynH1 plants were used as phenotypic data for QTL analysis. The seed set rates on the triploid hybrid plants varied from 0.07 to 0.72, with an average of 0.33 (Figure 6A). QTL analysis detected a major QTL for selfed seed level on chromosome 3B, between markers *Xgwm285* and *Xcfp1012*, with a logarithm of odds (LOD) score of 10.0 (Figure 6B). No segregation distortion was detected for the two markers with a genetic distance of 1 cM (Table S2; Figure 6B). Both markers are located in deletion bin 3BS5-0.07-0.33 (Paux *et al.* 2008). This QTL explained 29.8% of the phenotypic variance. The allele from LDN showed a positively additive effect and was responsible for the high frequency of genome doubling in hybrids. Besides this major QTL, we detected a weaker QTL between *Xgwm526* and *wPt-7175* on 2A chromosome with a LOD score of 3.4 and explained 8.7% of the phenotypic variance. However, the two markers covered a long genetic distance of 46.33 cM. We did not further analyze this QTL.

The effects of this QTL on 3B were further assessed in the second haploid population, SynH2 (LDN/AS2255//AS60). A linkage map for chromosome 3B was constructed using the flanking SSR markers of this QTL. The selfed seed set rates of the 89 triploid SynH2 plants varied from 0.03 to 0.57, with an average of 0.28 (Figure 6C). QTL analysis confirmed the existence of a major QTL that was located close to marker *Xcfp1012*, with a LOD score of 8.9 (Figure 6D). This QTL explained 38.4% of the phenotypic variation and, again, the allele from LDN showed a positively additive effect. However, this QTL showed a longer genetic distance from *Xcfp1012* in SynH2 (Figure 6C; Table S3) than SynH1 (Figure 6B; Table S2). The difference of the genetic distance between two mapping populations may be caused by smaller population or/and lower marker density in SynH2.

We also conducted a QTL analysis on doubled haploid populations SynDH1 and SynDH2. Their seed set rates were used in a QTL analysis. However, no QTL for seed set on chromosome 3B was found in either population (Figures 6, B and D).

### Identification of homologs of TAM/CYCA1;2 in wheat

Wheat chromosome 3B shows a high level of synteny to Brachypodium distachyon chromosome 2 and rice chromosome 1 (Paux *et al.* 2008; Qi *et al.* 2010; Shatalina *et al.* 2013). Sequence comparisons indicated that gene Os01g13260.1 on rice chromosome 1 and Bradi2g07946.1 on Brachypodium chromosome 2 are homologs of *tam*/*cyca1;2*, which is responsible for the formation of unreduced gametes in *Arabidopsis thaliana* (Magnard *et al.* 2001; Wang *et al.* 2004; d’Erfurth *et al.* 2010). This prompted us to identify the homologs of *tam* in wheat, designated as *Ttam*, where "T" represents "*Triticum*."

With a pair of primers designed from the CDS of Os01g13260.1, we cloned two highly similar sequences from LDN (Figure S1, between blue arrowheads). BLASTing them against rice (http://rice.plantbiology.msu.edu/analyses_search_blast.shtml), B. distachyon (http://www.brachypodium.org/gmod/alignment/blast_finders/new), and A. thaliana (http://rice.plantbiology.msu.edu/analyses_search_blast.shtml) sequences resulted in the best hits to Os01g13260.1, Bradi2g07946.1, and *tam*/*cyca1;2*, respectively. Therefore, we assumed that the two cloned wheat sequences were partial sequences of *Ttam*. We searched for similarity against the BAC-based assembled sequence of chromosome 3B of Chinese Spring (Choulet *et al.* 2014) and found that *Ttam*-*3B* was carried by a large scaffold of 612 kb corresponding to BAC contig ctg1014 (Paux *et al.* 2008) anchored to deletion bin 3BS1-0.33-0.55. Similarity search against the draft sequence of the A genome of *T. urartu* (Ling *et al.* 2013) revealed the presence of a homeologous copy in scaffold 25600, which belongs to deletion bin 3AS4-0.45-1.00. We failed to detect a homeologous copy on the D-genome draft sequence of *Ae. tauschii* (Jia *et al.* 2013).

**Figure 7:**
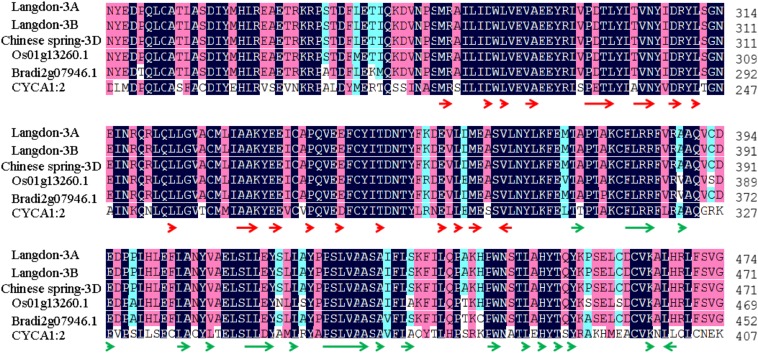
Amino acid comparison of TAM/CYCA1:2 among wheat (Langdon-3A, 3B, and Chinese Spring-3D), rice (Os01g13260.1), Brachypodium (Bradi2g07946.1), and Arabidopsis (CYCA1:2/TAM). Red and green arrowheads indicate the two cyclin domains in Arabidopsis (Marchler-Bauer and Bryant 2004; Marchler-Bauer *et al.* 2009, 2011).

Based on the scaffold sequence of ctg1014, a pair of primers was designed to clone the full *Ttam* CDS from *T. turgidum* LDN, AS313, AS2255, AS308, and PI14892, and common hexaploid wheat Chinese Spring. The F_1_ hybrids of these *T. turgidum* lines with Ae. tauschii showed a different abilities to produce functional gametes (Zhang *et al.* 2010). However, all the analyzed lines had the same *Ttam*-*3B* CDS sequence (GenBank no. KJ863558). This suggested that this gene has been relatively well-conserved. Similarly, the homeologous gene on 3A was the same among analyzed lines (GenBank no. KJ863557). Although there were some variations in the DNA sequences among *Ttam*-*3A*, *Ttam*-*3B*, and *Ttam*-*3D* (GenBank no. KJ863559) (Figure S1), their two cyclin domains were the same and were conserved among species, including Arabidopsis (Marchler-Bauer and Bryant 2004; Marchler-Bauer *et al.* 2009, 2011), rice, and Brachypodium (Figure 7).

**Figure 8:**
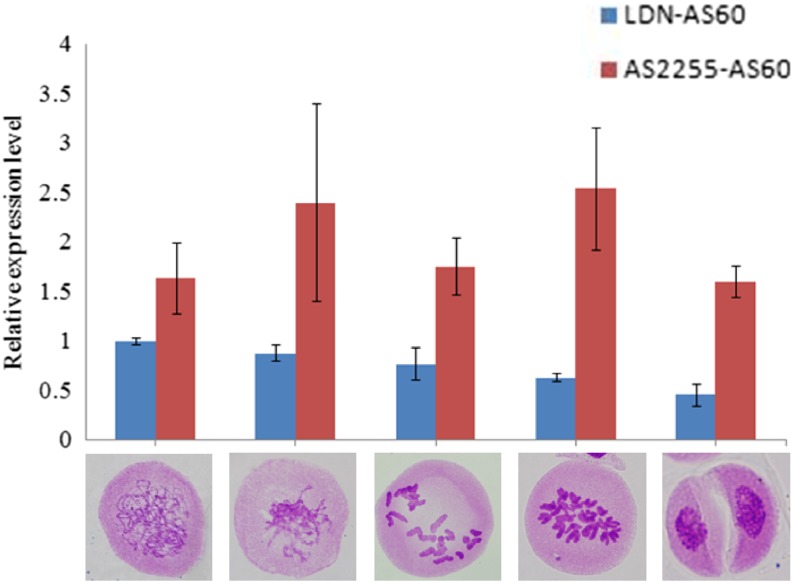
Expression changes of Ttam at different meiotic stages in the LDN×AS60 and AS2255×AS60 hybrids. Gene expression changes were assayed by qRT-PCR and analyzed by the 2*^−ΔΔCt^* method. Wheat β-actin was used as the reference gene.

We further checked the expression levels of Ttam at five meiotic stages, including leptotene to pachytene (LP), diplotene to diakinesis (DD), metaphase I, restitution nucleus, and dyads in *T. turgidum*×*Ae. tauschii* hybrids, with qRT-PCR. Overall, LDN-AS60 hybrids showed significantly lower expression levels than the AS2255×AS60 hybrids at all stages (Figure 8). Although the LDN-AS60 hybrids showed consistent expression among biological replicates in all stages, the AS2255-AS60 hybrids showed high variations in the first four stages, except at the dyad stage (Figure 8). This indicated that the expression of *Ttam* might be more sensitive to environmental influences in the AS2255-AS60 hybrid background than in the LDN-AS60 hybrids.

## Discussion

### First division restitution exhibited prolonged cell division during meiosis I

FDR predominated in LDN×AS60 hybrids, leading to dyad formation (Figure 4, A and B). Besides FDR, a large number of meiocytes in the AS313×AS60 and the AS2255×AS60 hybrids went through standard meiosis, which produced triads and tetrads (Figure 4, C and D). The coexistence of FDR and standard meiosis within an anther (Figure 4, C and D) suggested that the duration of formation of dyads by FDR (single cell division) and for the formation of triads or tetrads by standard meiosis (two cell divisions) were similar. Thus, compared with standard meiosis I, FDR produced a phenotype of prolonged cell division and the overwhelming superiority of dyads observed in the LDN×AS60 hybrids suggested that most PMCs did not enter into meiosis II, which allowed sufficient time to finish meiotic restitution and then to form dyads (Figure 4, A and B).

### Pathways of first division restitution

FDR occurs because of an equational division with segregation of sister chromatids of univalents before telophase I (Ramanna and Jacobsen 2003). Our data show that equational division in *T. turgidum*×*Ae. tauschii* hybrids occurs either by direct division of univalents aligned on the equator at MI (Figure 3, B and C), as reported by Matsuoka and Nasuda (2004) and Zhang *et al.* (2007), or by a pathway of forming restitution nuclei (Figure 3, D–F), as shown by Xu and Joppa (2000). The latter may be responsible for the higher hexaploidization capacity of LDN×AS60 (Figure 4A) compared with the AS2255×AS60 (Figure 4C) and the AS313×AS60 hybrids. This is because this pathway appears to be more effective in producing unreduced gametes than the former due to the fact that the formation of restitution nuclei effectively organizes all chromosomes together, allowing all chromosomes to divide equationally (Xu and Joppa 2000). FDR without restitution nuclei may explain the production of aneuploid gametes because it might not have organized all chromosomes together. As Oleszczuk and Lukaszewski (2014) recently illustrated, occasional pairing of homeologous chromosomes at MI, combined with sister chromatid division of univalents, generates aneuploids. Precocious MI migration to the poles by some undivided univalents also provides an additional source of aneuploid gametes.

The two FDR pathways can coexist in the same anther of *T. turgidum*×*Ae. tauschii* hybrids (Xu and Joppa 2000; Zhang *et al.* 2007) and are affected by the genetic backgrounds of the hybrids. Hybrids with a high capacity of ploidization, such as that between LDN and *Ae. tauschii*, show high FDR frequency with restitution nuclei (Figure 4A) (Xu and Joppa 2000; Matsuoka and Nasuda 2004; Matsuoka *et al.* 2013), while wide hybrids with low ploidization capacity, like rye hybrids with *T. turgidum* or *T. aestivum*, usually undergo FDR without restitution nuclei (Silkova *et al.* 2011, 2013; Oleszczuk and Lukaszewski 2014). Environmental sensitivity of meiotic restitution in wide hybrids has also been reported (Ramsey and Schemske 1998; Mable 2004; Brownfield and Kohler 2011; Pécrix *et al.* 2011; Mason *et al.* 2011). These studies suggest that gene expression level might be important in meiotic restitution.

### *QTug.sau-3B* is a haploid-dependent QTL for wheat hexaploidization

Selfed seeds of *T. turgidum*×*Ae. tauschii* haploid hybrids usually result from the union of unreduced gametes (Matsuoka *et al.* 2013), and the level of seed setting was therefore used as a measure of the production of functionally unreduced gametes. Besides QTL for unreduced gamete formation, however, genes that regulate other reproductive activities, such as fertilization and seed development in hybrids, may also affect the seed set. If the QTL identified in the triploid populations is responsible for the production of unreduced gametes, then this QTL should be nondetectable in the doubled haploid populations. This is due to the fact that unreduced gametes do not occur in normal hexaploid wheat in which all its chromosomes are paired as bivalents at MI (Ressurreição *et al.* 2012). Otherwise, if this QTL is responsible for other reproductive activities, then it should be detectable in a doubled haploid population. We tested the hypothesis by QTL analysis on doubled haploid populations SynDH1 and SynDH2, and no QTL for seed set on chromosome 3B was found in either population (Figures 6, B and D). This proved that the haploid-dependent QTL is responsible for the unreduced gametes formation or, as De Storme and Geelen (2013) suggested, for the observed "univalent-dependent meiotic nonreduction." Hence, we designated the QTL as *QTug.sau*-*3B* (following guidelines of McIntosh *et al.* 2011), where T represents Triticum, ug represents unreduced gametes, and sau represents Sichuan Agricultural University.

### *TAM* and *QTug.sau-3B* may be orthologous

*QTug.sau-3B* is a syntenic TAM locus in rice and Brachypodium. In Arabidopsis, *TAM* encodes *CYCA1;2*. A weak mutant tam-1 exhibits a phenotype of delayed asynchronous cell divisions during male meiosis in Arabidopsis, although it, like the wild-type, does eventually produce haploid gametes (Magnard *et al.* 2001; Wang *et al.* 2004). Changes in cell-cycle progression in tam-1 are influenced by temperature. However, stronger tam mutants abolish the second meiotic division and systematically produce unreduced gametes (d’Erfurth *et al.* 2010; Bulankova *et al.* 2010, 2013). Combining the tam-2 mutation with the Atspoll-1 mutation that eliminates recombination and the Atrec8 mutation that ensures segregation of sister chromatids rather than homologs converts meiosis into a mitosis-like division (d’Erfurth *et al.* 2010). This tam-2/Atspoll-1/Atrec8 triple mutant (also called MiMe-2) displays phenotypes very similar to those observed in the LDN×AS60 hybrids in several ways. First, unpaired chromosomes or univalents align on the MI plate and sister chromatids separate at anaphase I (Figure 3). Second, they mostly produce dyads due to the absence of the second meiotic division (Figure 4B). Third, both aneuploids at low frequencies and euploids exist in the selfed progeny. The similarity in the phenotypic effects and their syntenic relationships suggest that *TAM* and *QTug.sau*-*3B* may be orthologous.

### Expression of *Ttam* is reduced in hybrids with high hexaploidization capacity

Complexes formed by cyclins and cyclin-dependent kinases (CDK) are essential for progression through meiotic cell cycles. The transition from meiosis I to meiosis II requires a fine balance of cyclin/CDK activity in that it must be sufficiently low to exit meiosis I while being maintained at a level sufficient to promote entry into meiosis II (Marston and Amon 2004). In Arabidopsis, tam mutants cause a moderate decrease of cyclin/CDK activity, which leads to the failure of the meiosis I–meiosis II transition but does not impair the prophase to meiosis I transition, resulting in meiotic restitution and the production of unreduced gametes (d’Erfurth *et al.* 2010).

Wheat homologs of *tam* (*Ttam*) are located in homeologous group 3. Our data show that *Ttam* expression was significantly lower in LDN×AS60 hybrids than in AS2255×AS60 hybrids at all of the five stages we analyzed (Figure 8). Reduced Ttam expression could be the cause of the higher frequency of unreduced gametes in LDN×AS60 hybrids than in AS2255×AS60 hybrids. However, we failed to differentiate *Ttam-3B* expression from that of its homeologous 3A and 3D alleles by PCR amplification because of their sequence similarity. Although LDN and AS2255 have the same CDS at Ttam-3B, their expression levels might be different. Our study did not determine a relationship between *Ttam-3B* and *QTug.sau-3B*. QTug.sau-3B may be a cis regulatory factor of *Ttam-3B* or a trans regulatory factor of *Ttam-3A* or *Ttam-3D* affecting Ttam expression. Alternatively, *QTug.sau-3B* may be the same as *Ttam-3B*, and may be specifically regulated by a factor or factors in other chromosome regions.

Compared with the LDN×AS60 hybrids, the lower frequency of unreduced gametes and more asynchronous cell cycles in AS2255×AS60 hybrids could be caused by higher expression of Ttam genes in these hybrids (Figure 8). Meiocytes with a relatively low expression could undergo FDR, while those with high expression may undergo standard meiosis (d’Erfurth *et al.* 2010). The coexistence of FDR and standard meiosis within an anther suggests the existence of variable expression among meiocytes (Figure 4, C and D). Perhaps some cells within an anther have higher residual TAM activity than others and are thus able to enter the next cell cycle earlier than other cells. This may cause asynchronous cell cycles (Figure 4, A and C and Figure 5). This hypothesis is supported by our observation that adjacent cells in an anther seem to be more synchronous than those that are not close to each other (Figure 4A and Figure 5). It is possible that adjacent cells have similar TAM concentrations.

## Supplementary Material

Supporting Information
