# Supplementary material for: QTug.sau-3B Is a Major Quantitative Trait Locus for Wheat Hexaploidization
Source: G3 (Bethesda). 2014 Aug 15;4(10):1943–53. doi: 10.1534/g3.114.013078 (PMC4199700; doi:10.1534/g3.114.013078)
Supplement: Supporting Information [file supp_4_10_1943__index.html]

QTug.sau-3B Is a Major Quantitative Trait Locus for Wheat Hexaploidization — Supporting Information 

# *QTug.sau-3B* Is a Major Quantitative Trait Locus for Wheat Hexaploidization

## Supporting Information for Hao *et al.*, 2014

**Files in this Data Supplement:**

- Supporting Information - Figure S1 and Tables S1-S4 (PDF, 2.2 MB)
- Table S1 - Primers used in this study (PDF, 86 KB)
- Table S4 - GenBank no. KJ863557 organism=Triticum turgidum T. turgidum Langdon Ttam-3A mRNA , complete cds; GenBank no. KJ863558 organism=Triticum turgidum T. turgidum Langdon Ttam-3B mRNA, complete cds; GenBank no. KJ863559 organism=Triticum aestivum T. aestivum Chinese spring Ttam-3D mRNA, complete cds (PDF, 58 KB)
- Figure S1 - Coding DNA sequence (CDS) comparisons among homologs of tam in wheat chromosomes 3A, 3B and 3D. Primer sequences for the partial and full CDS are indicated by blue and green arrowheads, respectively. The bars in the major line indicate the SNPs. (TIFF, 3.1 MB)
- Table S2 - Genotypes, phenotypes and linkage map for DH1 population (.xls, 495 KB)
- Table S3 - Genotypes, phenotypes and linkage map for DH2 population (.xls, 31 KB)
