# Supplementary material for: QTug.sau-3B Is a Major Quantitative Trait Locus for Wheat Hexaploidization
Source: G3 (Bethesda). 2014 Aug 15;4(10):1943–53. doi: 10.1534/g3.114.013078 (PMC4199700; doi:10.1534/g3.114.013078)
Supplement: Supporting Information [file supp_g3.114.013078_013078SI.pdf]

G3 · Genes | Genomes | Genetics

QTug.sau-3B is a major quantitative trait locus for wheat hexaploidization

Ming Hao [*, 1], Jiangtao Luo [*, 1], Deying Zeng [*], Li Zhang[§], Shunzong Ning [*], Zhongwei Yuan [*], Zehong Yan [*], Huaigang Zhang [†], Youliang Zheng [*], Catherine Feuillet [‡, 2], Frédéric Choulet [‡], Yang Yen [**], Lianquan Zhang [*], Dengcai Liu [*, †, 3]

[*] Triticeae Research Institute, Sichuan Agricultural University at Chengdu, Wenjiang, Sichuan 611130, P.R. China

[§]Institute of Ecological Forestry, Sichuan Agricultural University at Chengdu, Wenjiang, Sichuan 611130, P.R. China

[†] Key Laboratory of Adaptation and Evolution of Plateau Biota, Northwest Institute of Plateau Biology, Chinese Academy of Sciences, Xining 810001, P.R. China

[‡]INRA University Blaise Pascal, Joint Research Unit 1095 Genetics Diversity and Ecophysiology of Cereals, Clermont-Ferrand 63039, France

[**] Department of Biology and Microbiology, South Dakota State University, Brookings, SD 57007, USA

[1]The authors contributed equal to this study.

[2] Present address: Bayer, Research & Development, 3500 Paramount Parkway, Morrisville, NC 27560, USA

DOI: 10.1534/g3.114.013078

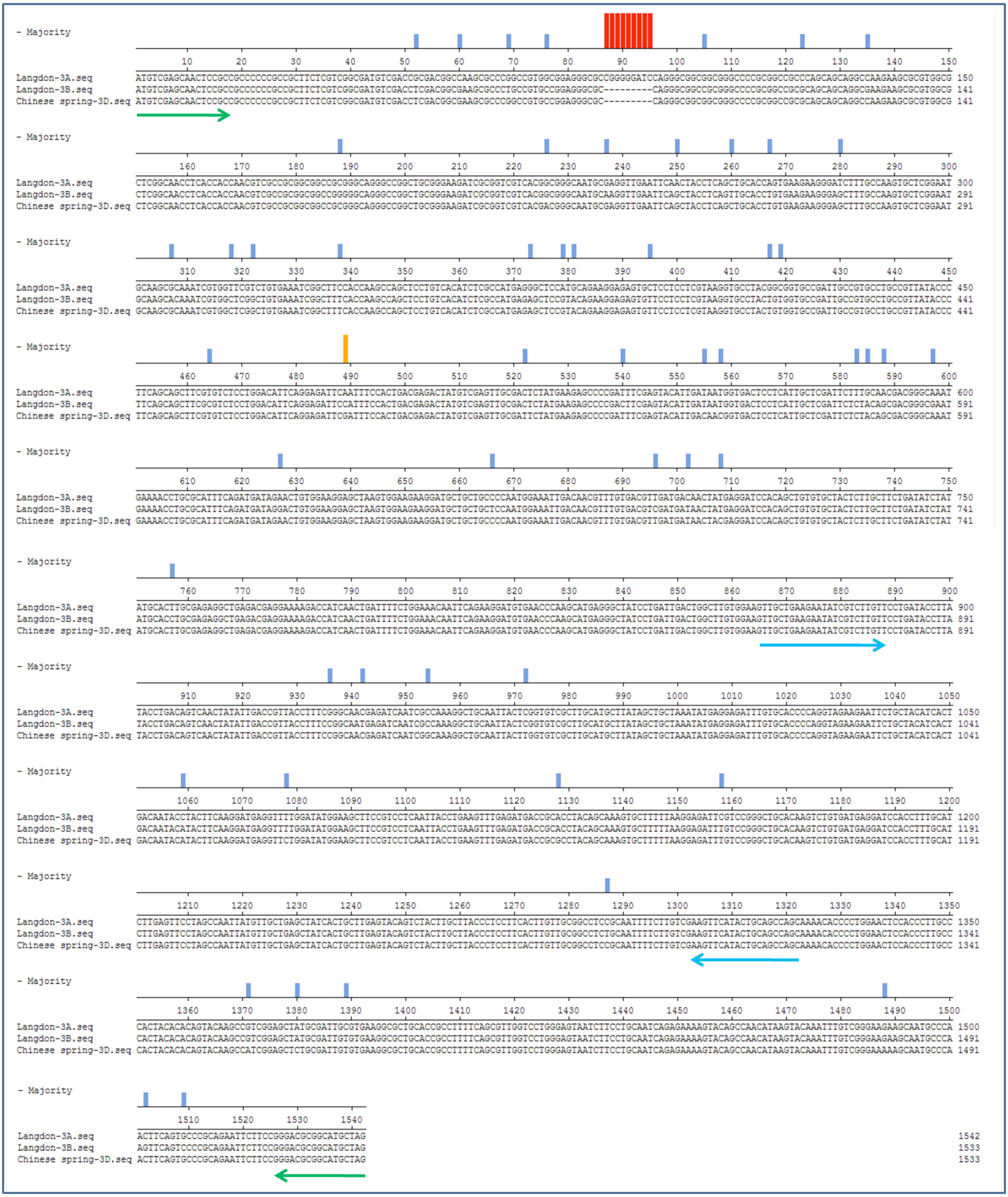

Figure S1 Coding DNA sequence (CDS) comparisons among homologs of *tam* in wheat chromosomes 3A, 3B and 3D. Primer sequences for the partial and full CDS are indicated by blue and green arrowheads, respectively. The bars in the major line indicate the SNPs.

Table S1 Primers used in this study

Name of primer	Primer sequences (5'→3')	Annealing temperature
6C6-3-F1	CTACTTCCACTGCACCAGAC	60
6C6-3-R1	CGCCCTACTTTGCACACAAAA	
F1	GTCGCTGAAGAATATCGTCTTGTT	58
R1	TGTTGGCTGCAGTATGAATTT	
F2	ATGTCGAGCAACTCCGC	60
R2	CTAGCATGCCGCGTCCC	
F10-RT	GCTTACCCTCCTTCACTTGT	60
R10-RT	CCTTCACGCAATCGCATAG	

Table S2 Genotypes, phenotypes and linkage map for DH1 population.

Table S3 Genotypes, phenotypes and linkage map for DH2 population.

Available for download at
http://www.g3journal.org/lookup/suppl/doi:10.1534/g3.114.013078/-/DC1

> GenBank no. KJ863557 [organism=*Triticum turgidum*] *T. turgidum* Langdon Ttam-3A mRNA , complete cds

ATGTCGAGCAACTCCGCCGCCCCCGCCGCTTCTCGTCGGCGATGTCGACCGCGACGG
CCAAGCGCCCGGCCGTGGCGGAGGGCGCCGGGGGATCCAGGGCGGCGGCGGCCCCC
GCGGCCGCCCAGCAGCAGGCCAAGAAGCGCGTGGCGCTCGGCAACCTCACCACCAAC
GTCGCCGCGGCGGCCGCGGGCAGGGCCGGCTGCGGGAAGATCGCGGTCGTCACGGC
GGGCAATGCGAGGTTGAATTCAACTACCTCAGCTGCACCAGTGAAGAAGGGATCTTT
GCCAAGTGCTCGGAATGCAAGCGCAAATCGTGGTTCGTCTGTGAAATCGGCTTCCAC
CAAGCCAGCTCCTGTCACATCTCGCCATGAGGGCTCCATGCAGAAGGAGAGTGCTCC
TCCTCGTAAGGTGCCTACGGCGGTGCCGATTGCCGTGCCTGCCGTTATACCCTTCAGC
AGCTTCGTGTCTCCTGGACATTCAGGAGATTCAATTTCCACTGACGAGACTATGTCGA
GTTGCGACTCTATGAAGAGCCCCGATTTCGAGTACATTGATAATGGTGACTCCTCATT
GCTCGATTCTTTGCAACGACGGGCAAATGAAAACCTGCGCATTTCAGATGATAGAAC
TGTGGAAGGAGCTAAGTGGAAGAAGGATGCTGCTGCCCCAATGGAAATTGACAACGT
TTGTGACGTTGATGACAACTATGAGGATCCACAGCTGTGTGCTACTCTTGCTTCTGAT
ATCTATATGCACTTGCGAGAGGCTGAGACGAGGAAAAGACCATCAACTGATTTTCTG
GAAACAATTCAGAAGGATGTGAACCCAAGCATGAGGGCTATCCTGATTGACTGGCTT
GTGGAAGTTGCTGAAGAATATCGTCTTGTTCCTGATACCTTATACCTGACAGTCAACT
ATATTGACCGTTACCTTTCGGGCAACGAGATCAATCGCCAAAGGCTGCAATTACTCGG
TGTCGCTTGCATGCTTATAGCTGCTAAATATGAGGAGATTTGTGCACCCCAGGTAGAA
GAATTCTGCTACATCACTGACAATACCTACTTCAAGGATGAGGTTTTGGATATGGAAG
CTTCCGTCCTCAATTACCTGAAGTTTGAGATGACCGCACCTACAGCAAAGTGCTTTTT
AAGGAGATTCGTCCGGGCTGCACAAGTCTGTGATGAGGATCCACCTTTGCATCTTGAG
TTCCTAGCCAATTATGTTGCTGAGCTATCACTGCTTGAGTACAGTCTACTTGCTTACCC
TCCTTCACTTGTTGCGGCCTCCGCAATTTTCTTGTCGAAGTTCATACTGCAGCCAGCAA
AACACCCCTGGAACTCCACCCTTGCCCACTACACACAGTACAAGCCGTCGGAGCTAT
GCGATTGCGTGAAGGCGCTGCACCGCCTTTTCAGCGTTGGTCCTGGGAGTAATCTTCC
TGCAATCAGAGAAAAGTACAGCCAACATAAGTACAAATTTGTCGGGAAGAAGCAAT
GCCCAACTTCAGTGCCCGCAGAATTCTTCCGGGACGCGGCATGCTAG

> GenBank no. KJ863558 [organism=*Triticum turgidum*] *T. turgidum* Langdon Ttam-3B mRNA, complete cds

ATGTCGAGCAACTCCGCCGCCCCCGCCGCTTCTCGTCGGCGATGTCGACCTCGACGG
CGAAGCGCCCTGCCGTGCCGGAGGGCGCCAGGGCGGCCGCGGGCCCCGCGGCCGCG
CAGCAGCAGGCGAAGAAGCGCGTGGCGCTCGGCAACCTCACCACCAACGTCGCCGCG
GCGGCCGGGGGCAGGGCCGGCTGCGGGAAGATCGCGGTCGTCACGGCGGGCAATGC
AAGGTTGAATTCAGCTACCTCAGTTGCACCTGTGAAGAAGGGAGCTTTGCCAAGTGC
TCGGAATGCAAGCACAAATCGTGGCTCGGCTGTGAAATCGGCTTTCACCAAGCCAGC
TCCTGTCACATCTCGCCATGAGAGCTCCGTACAGAAGGAGAGTGTTCCTCCTCGTAAG
GTGCCTACTGTGGTGCCGATTGCCGTGCCTGCCGTTATACCCTTCAGCAGCTTCGCGT
CTCCTGGACATTCAGGAGATTCCATTTCCACTGACGAGACTATGTCGAGTTGCGACTC
TATGAAGAGCCCCGACTTCGAGTACATTGATAATGGTGACTCCTCATTGCTCGATTCT

CTACAGCGACGGGCGAATGAAAACCTGCGCATTTCAGATGATAGGACTGTGGAAGGA
GCTAAGTGGAAGAAGGATGCTGCTGCTCCAATGGAAATTGACAACGTTTGTGACGTC
GATGATAACTATGAGGATCCACAGCTGTGTGCTACTCTTGCTTCTGATATCTATATGC
ACCTGCGAGAGGCTGAGACGAGGAAAAGACCATCAACTGATTTTCTGGAAACAATTC
AGAAGGATGTGAACCCAAGCATGAGGGCTATCCTGATTGACTGGCTTGTGGAAGTTG
CTGAAGAATATCGTCTTGTTCCTGATACCTTATACCTGACAGTCAACTATATTGACCG
TTACCTTTCCGGCAATGAGATCAATCGCCAAAGGCTGCAATTACTCGGTGTCGCTTGC
ATGCTTATAGCTGCTAAATATGAGGAGATTTGTGCACCCCAGGTAGAAGAATTCTGCT
ACATCACTGACAATACATACTTCAAGGATGAGGTTTTGGATATGGAAGCTTCCGTCCT
CAATTACCTGAAGTTTGAGATGACCGCACCTACAGCAAAGTGCTTTTTAAGGAGATTT
GTCCGGGCTGCACAAGTCTGTGATGAGGATCCACCTTTGCATCTTGAGTTCCTAGCCA
ATTATGTTGCTGAGCTATCACTGCTTGAGTACAGTCTACTTGCTTACCCTCCTTCACTT
GTTGCGGCCTCTGCAATTTTCTTGTCGAAGTTCATACTGCAGCCAGCAAAACACCCCT
GGAACTCCACCCTTGCCCACTACACACAGTACAAGCCGTCGGAGCTATGCGATTGTGT
GAAGGCGCTGCACCGCCTTTTCAGCGTTGGTCCTGGGAGTAATCTTCCTGCAATCAGA
GAAAAGTACAGCCAACATAAGTACAAATTTGTCGGGAAGAAGCAATGCCCAAGTTCA
GTCCCCGCAGAATTCTTCCGGGACGCGGCATGCTAG

> GenBank no. KJ863559 [organism=*Triticum aestivum*] *T. aestivum* Chinese spring Ttam-3D mRNA, complete cds

ATGTCGAGCAACTCCGCCGCCCCCCGCCGCTTCTCGTCGGCGATGTCGACCTCGACGG
CGAAGCGCCCGGCCGTGCCGGAGGGCGCCAGGGCGGCGGCGGGCCCCGCGGCCGCG
CAGCAGCAGGCCAAGAAGCGCGTGGCGCTCGGCAACCTCACCACCAACGTCGCCGCG
GCGGCCGCGGGCAGGGCCGGCTGCGGGAAGATCGCGGTCGTCACGACGGGCAATGC
GAGGTTGAATTCAGCTACCTCAGCTGCACCTGTGAAGAAGGGAGCTTTGCCAAGTGC
TCGGAATGCAAGCGCAAATCGTGGCTCGGCTGTGAAATCGGCTTTCACCAAGCCAGC
TCCTGTCACATCTCGCCATGAGAGCTCCGTACAGAAGGAGAGTGTTCCTCCTCGTAAG
GTGCCTACTGTGGTGCCGATTGCCGTGCCTGCCGTTATACCCTTCAGCAGCTTCGTGT
CTCCTGGACATTCAGGAGATTCGATTTCCACTGACGAGACTATGTCGAGTTGCGATTC
TATGAAGAGCCCCGATTTCGAGTACATTGACAACGGTGACTCCTCATTGCTCGATTCT
CTACAGCGACGGGCAAATGAAAACCTGCGCATTTCAGATGATAGAACTGTGGAAGGA
GCTAAGTGGAAGAAGGATGCTGCTGCCCCAATGGAAATTGACAACGTTTGTGACGTT
GATGATAACTACGAGGATCCACAGCTGTGTGCTACTCTTGCTTCTGATATCTATATGC
ACTTGCGAGAGGCTGAGACGAGGAAAAGACCATCAACTGATTTTCTGGAAACAATTC
AGAAGGATGTGAACCCAAGCATGAGGGCTATCCTGATTGACTGGCTTGTGGAAGTTG
CTGAAGAATATCGTCTTGTTCCTGATACCTTATACCTGACAGTCAACTATATTGACCG
TTACCTTTCCGGCAACGAGATCAATCGGCAAAGGCTGCAATTACTTGGTGTCGCTTGC
ATGCTTATAGCTGCTAAATATGAGGAGATTTGTGCACCCCAGGTAGAAGAATTCTGCT
ACATCACTGACAATACATACTTCAAGGATGAGGTTCTGGATATGGAAGCTTCCGTCCT
CAATTACCTGAAGTTTGAGATGACCGCGCCTACAGCAAAGTGCTTTTTAAGGAGATTT
GTCCGGGCTGCACAAGTCTGTGATGAGGATCCACCTTTGCATCTTGAGTTCCTAGCCA
ATTATGTTGCTGAGCTATCACTGCTTGAGTACAGTCTACTTGCTTACCCTCCTTCACTT
GTTGCGGCCTCCGCAATTTTCTTGTCGAAGTTCATACTGCAGCCAGCAAAACACCCCT

GGAACTCCACCCTTGCCCACTACACACAGTACAAGCCATCGGAGCTCTGCGATTGTGT
GAAGGCGCTGCACCGCCTTTTCAGCGTTGGTCCTGGGAGTAATCTTCCTGCAATCAGA
GAAAAGTACAGCCAACATAAGTACAAATTTGTCGGGAAAAAGCAATGCCCAACTTCA
GTGCCCGCAGAATTCTTCCGGGACGCGGCATGCTAG
