# Supplementary material for: QTug.sau-3B Is a Major Quantitative Trait Locus for Wheat Hexaploidization
Source: G3 (Bethesda). 2014 Aug 15;4(10):1943–53. doi: 10.1534/g3.114.013078 (PMC4199700; doi:10.1534/g3.114.013078)
Supplement: Supporting Information [file supp_g3.114.013078_TableS1.pdf]

Table S1 Primers used in this study

Name of primer	Primer sequences (5'→3')	Annealing temperature
6C6-3-F1	CTACTTCCACTGCACCAGAC	60
6C6-3-R1	CGCCCTACTTTGCACACAAAA	
F1	GTCGCTGAAGAATATCGTCTTGTT	58
R1	TGTTGGCTGCAGTATGAATTT	
F2	ATGTCGAGCAACTCCGC	60
R2	CTAGCATGCCGCGTCCC	
F10-RT	GCTTACCCTCCTTCACTTGT	60
R10-RT	CCTTCACGCAATCGCATAG	
