# Supplementary material for: QTug.sau-3B Is a Major Quantitative Trait Locus for Wheat Hexaploidization
Source: G3 (Bethesda). 2014 Aug 15;4(10):1943–53. doi: 10.1534/g3.114.013078 (PMC4199700; doi:10.1534/g3.114.013078)
Supplement: Supporting Information [file supp_g3.114.013078_TableS4.pdf]

> GenBank no. KJ863557 [organism=*Triticum turgidum*] *T. turgidum* Langdon Ttam-3A mRNA , complete cds

ATGTCGAGCAACTCCGCCGCCCCCCGCCGCTTCTCGTCGGCGATGTCGACCGCGACGG
CCAAGCGCCCGGCCGTGGCGGAGGGCGCCGGGGGATCCAGGGCGGCGGCGGGCCCC
GCGGCCGCCCAGCAGCAGGCCAAGAAGCGCGTGGCGCTCGGCAACCTCACCACCAAC
GTCGCCGCGGCGGCCGCGGGCAGGGCCGGCTGCGGGAAGATCGCGGTCGTCACGGC
GGGCAATGCGAGGTTGAATTCAACTACCTCAGCTGCACCAGTGAAGAAGGGATCTTT
GCCAAGTGCTCGGAATGCAAGCGCAAATCGTGGTTCGTCTGTGAAATCGGCTTCCAC
CAAGCCAGCTCCTGTCACATCTCGCCATGAGGGCTCCATGCAGAAGGAGAGTGCTCC
TCCTCGTAAGGTGCCTACGGCGGTGCCGATTGCCGTGCCTGCCGTTATACCCTTCAGC
AGCTTCGTGTCTCCTGGACATTCAGGAGATTCAATTTCCACTGACGAGACTATGTCGA
GTTGCGACTCTATGAAGAGCCCCGATTTCGAGTACATTGATAATGGTGACTCCTCATT
GCTCGATTCTTTGCAACGACGGGCAAATGAAAACCTGCGCATTTCAGATGATAGAAC
TGTGGAAGGAGCTAAGTGGAAGAAGGATGCTGCTGCCCCAATGGAAATTGACAACGT
TTGTGACGTTGATGACAACTATGAGGATCCACAGCTGTGTGCTACTCTTGCTTCTGAT
ATCTATATGCACTTGCGAGAGGCTGAGACGAGGAAAAGACCATCAACTGATTTTCTG
GAAACAATTCAGAAGGATGTGAACCCAAGCATGAGGGCTATCCTGATTGACTGGCTT
GTGGAAGTTGCTGAAGAATATCGTCTTGTTCCTGATACCTTATACCTGACAGTCAACT
ATATTGACCGTTACCTTTCGGGCAACGAGATCAATCGCCAAAGGCTGCAATTACTCGG
TGTCGCTTGCATGCTTATAGCTGCTAAATATGAGGAGATTTGTGCACCCCAGGTAGAA
GAATTCTGCTACATCACTGACAATACCTACTTCAAGGATGAGGTTTTGGATATGGAAG
CTTCCGTCCTCAATTACCTGAAGTTTGAGATGACCGCACCTACAGCAAAGTGCTTTTT
AAGGAGATTCGTCCGGGCTGCACAAGTCTGTGATGAGGATCCACCTTTGCATCTTGAG
TTCCTAGCCAATTATGTTGCTGAGCTATCACTGCTTGAGTACAGTCTACTTGCTTACCC
TCCTTCACTTGTTGCGGCCTCCGCAATTTTCTTGTCGAAGTTCATACTGCAGCCAGCAA
AACACCCCTGGAACTCCACCCTTGCCCACTACACACAGTACAAGCCGTCGGAGCTAT
GCGATTGCGTGAAGGCGCTGCACCGCCTTTTCAGCGTTGGTCCTGGGAGTAATCTTCC
TGCAATCAGAGAAAAGTACAGCCAACATAAGTACAAATTTGTCGGGAAGAAGCAAT
GCCCAACTTCAGTGCCCGCAGAATTCTTCCGGGACGCGGCATGCTAG

> GenBank no. KJ863558 [organism=*Triticum turgidum*] *T. turgidum* Langdon Ttam-3B mRNA, complete cds

ATGTCGAGCAACTCCGCCGCCCCCCGCCGCTTCTCGTCGGCGATGTCGACCTCGACGG
CGAAGCGCCCTGCCGTGCCGGAGGGCGCCAGGGCGGCCGCGGGCCCCGCGGCCGCG
CAGCAGCAGGCGAAGAAGCGCGTGGCGCTCGGCAACCTCACCACCAACGTCGCCGCG
GCGGCCGGGGGCAGGGCCGGCTGCGGGAAGATCGCGGTCGTCACGGCGGGCAATGC
AAGGTTGAATTCAGCTACCTCAGTTGCACCTGTGAAGAAGGGAGCTTTGCCAAGTGC
TCGGAATGCAAGCACAAATCGTGGCTCGGCTGTGAAATCGGCTTTCACCAAGCCAGC
TCCTGTCACATCTCGCCATGAGAGCTCCGTACAGAAGGAGAGTGTTCCTCCTCGTAAG
GTGCCTACTGTGGTGCCGATTGCCGTGCCTGCCGTTATACCCTTCAGCAGCTTCGCGT
CTCCTGGACATTCAGGAGATTCCATTTCCACTGACGAGACTATGTCGAGTTGCGACTC
TATGAAGAGCCCCGACTTCGAGTACATTGATAATGGTGACTCCTCATTGCTCGATTCT

CTACAGCGACGGGCGAATGAAAACCTGCGCATTTCAGATGATAGGACTGTGGAAGGA
GCTAAGTGGAAGAAGGATGCTGCTGCTCCAATGGAAATTGACAACGTTTGTGACGTC
GATGATAACTATGAGGATCCACAGCTGTGTGCTACTCTTGCTTCTGATATCTATATGC
ACCTGCGAGAGGCTGAGACGAGGAAAAGACCATCAACTGATTTTCTGGAAACAATTC
AGAAGGATGTGAACCCAAGCATGAGGGCTATCCTGATTGACTGGCTTGTGGAAGTTG
CTGAAGAATATCGTCTTGTTCCTGATACCTTATACCTGACAGTCAACTATATTGACCG
TTACCTTTCCGGCAATGAGATCAATCGCCAAAGGCTGCAATTACTCGGTGTCGCTTGC
ATGCTTATAGCTGCTAAATATGAGGAGATTTGTGCACCCCAGGTAGAAGAATTCTGCT
ACATCACTGACAATACATACTTCAAGGATGAGGTTTTGGATATGGAAGCTTCCGTCCT
CAATTACCTGAAGTTTGAGATGACCGCACCTACAGCAAAGTGCTTTTTAAGGAGATTT
GTCCGGGCTGCACAAGTCTGTGATGAGGATCCACCTTTGCATCTTGAGTTCCTAGCCA
ATTATGTTGCTGAGCTATCACTGCTTGAGTACAGTCTACTTGCTTACCCTCCTTCACTT
GTTGCGGCCTCTGCAATTTTCTTGTCGAAGTTCATACTGCAGCCAGCAAAACACCCCT
GGAACTCCACCCTTGCCCACTACACACAGTACAAGCCGTCGGAGCTATGCGATTGTGT
GAAGGCGCTGCACCGCCTTTTCAGCGTTGGTCCTGGGAGTAATCTTCCTGCAATCAGA
GAAAAGTACAGCCAACATAAGTACAAATTTGTCGGGAAGAAGCAATGCCCAAGTTCA
GTCCCCGCAGAATTCTTCCGGGACGCGGCATGCTAG

> GenBank no. KJ863559 [organism=*Triticum aestivum*] *T. aestivum* Chinese spring Ttam-3D mRNA, complete cds

ATGTCGAGCAACTCCGCCGCCCCCCGCCGCTTCTCGTCGGCGATGTCGACCTCGACGG
CGAAGCGCCCGGCCGTGCCGGAGGGCGCCAGGGCGGCGGCGGGCCCCGCGGCCGCG
CAGCAGCAGGCCAAGAAGCGCGTGGCGCTCGGCAACCTCACCACCAACGTCGCCGCG
GCGGCCGCGGGCAGGGCCGGCTGCGGGAAGATCGCGGTCGTCACGACGGGCAATGC
GAGGTTGAATTCAGCTACCTCAGCTGCACCTGTGAAGAAGGGAGCTTTGCCAAGTGC
TCGGAATGCAAGCGCAAATCGTGGCTCGGCTGTGAAATCGGCTTTCACCAAGCCAGC
TCCTGTCACATCTCGCCATGAGAGCTCCGTACAGAAGGAGAGTGTTCCTCCTCGTAAG
GTGCCTACTGTGGTGCCGATTGCCGTGCCTGCCGTTATACCCTTCAGCAGCTTCGTGT
CTCCTGGACATTCAGGAGATTCGATTTCCACTGACGAGACTATGTCGAGTTGCGATTC
TATGAAGAGCCCCGATTTCGAGTACATTGACAACGGTGACTCCTCATTGCTCGATTCT
CTACAGCGACGGGCAAATGAAAACCTGCGCATTTCAGATGATAGAACTGTGGAAGGA
GCTAAGTGGAAGAAGGATGCTGCTGCCCCAATGGAAATTGACAACGTTTGTGACGTT
GATGATAACTACGAGGATCCACAGCTGTGTGCTACTCTTGCTTCTGATATCTATATGC
ACTTGCGAGAGGCTGAGACGAGGAAAAGACCATCAACTGATTTTCTGGAAACAATTC
AGAAGGATGTGAACCCAAGCATGAGGGCTATCCTGATTGACTGGCTTGTGGAAGTTG
CTGAAGAATATCGTCTTGTTCCTGATACCTTATACCTGACAGTCAACTATATTGACCG
TTACCTTTCCGGCAACGAGATCAATCGGCAAAGGCTGCAATTACTTGGTGTCGCTTGC
ATGCTTATAGCTGCTAAATATGAGGAGATTTGTGCACCCCAGGTAGAAGAATTCTGCT
ACATCACTGACAATACATACTTCAAGGATGAGGTTCTGGATATGGAAGCTTCCGTCCT
CAATTACCTGAAGTTTGAGATGACCGCGCCTACAGCAAAGTGCTTTTTAAGGAGATTT
GTCCGGGCTGCACAAGTCTGTGATGAGGATCCACCTTTGCATCTTGAGTTCCTAGCCA
ATTATGTTGCTGAGCTATCACTGCTTGAGTACAGTCTACTTGCTTACCCTCCTTCACTT
GTTGCGGCCTCCGCAATTTTCTTGTCGAAGTTCATACTGCAGCCAGCAAAACACCCCT

GGAACTCCACCCTTGCCCACTACACACAGTACAAGCCATCGGAGCTCTGCGATTGTGT
GAAGGCGCTGCACCGCCTTTTCAGCGTTGGTCCTGGGAGTAATCTTCCTGCAATCAGA
GAAAAGTACAGCCAACATAAGTACAAATTTGTCGGGAAAAAGCAATGCCCAACTTCA
GTGCCCGCAGAATTCTTCCGGGACGCGGCATGCTAG
