# Supplementary figures and images for: QTug.sau-3B Is a Major Quantitative Trait Locus for Wheat Hexaploidization
Source: G3 (Bethesda). 2014 Aug 15;4(10):1943–53. doi: 10.1534/g3.114.013078 (PMC4199700; doi:10.1534/g3.114.013078)

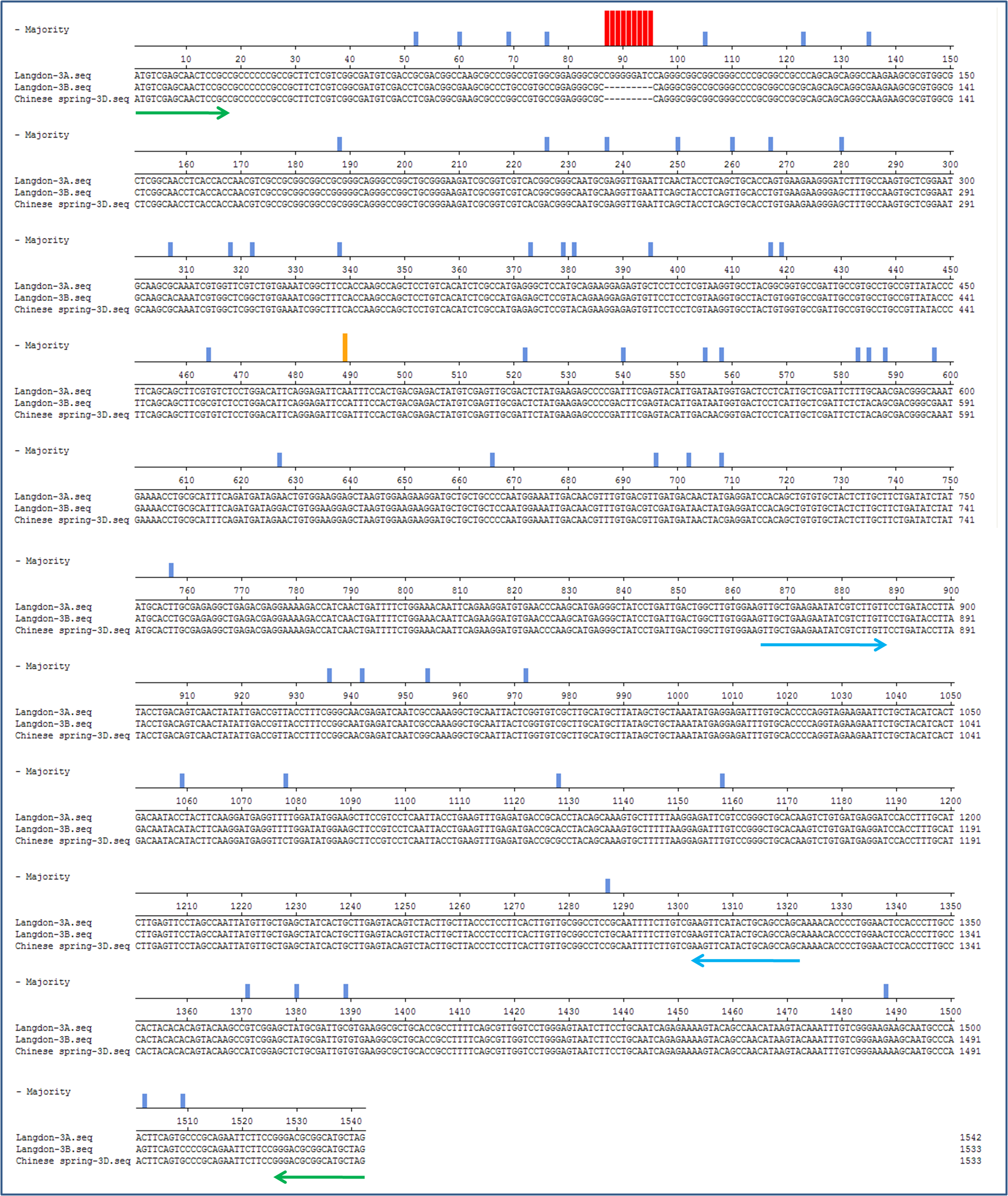

Supplement: Supporting Information [file supp_g3.114.013078_FigureS1.tif]
